# Regulation of Spontaneous Contractions in Intact Rat Bladder Strips and the Effects of Hydrogen Peroxide

**DOI:** 10.1155/2018/2925985

**Published:** 2018-02-04

**Authors:** Mingshuai Wang, Nianzeng Xing, Liyang Wu, Wen-Chu Huang, Zhenqun Xu, Guiming Liu

**Affiliations:** ^1^Department of Surgery, MetroHealth Medical Center, Case Western Reserve University, Cleveland, OH, USA; ^2^Department of Urology, Beijing Chao-Yang Hospital, Capital Medical University, Beijing, China; ^3^Division of Urogynecology, Department of Obstetrics and Gynecology, Mackay Memorial Hospital, Taipei, Taiwan; ^4^Department of Urology, Shengjing Hospital, China Medical University, Shenyang, China

## Abstract

Enhanced spontaneous contractions are associated with overactive bladder. Elevated levels of reactive oxygen species might contribute to enhanced spontaneous contractions. We investigated the regulation of spontaneous contractions and the effects of hydrogen peroxide (H_2_O_2_) in intact rat bladder strips. The spontaneous contractions were measured using a tissue bath system. The vehicle or the specific activators/blockers were applied and followed by the application of 0.003 g% H_2_O_2_. The basal tension, amplitude, and frequency of spontaneous contractions were quantified. Nisoldipine and bisindolylmaleimide 1 had no effects on spontaneous contractions. SKF96365 and Y27632 decreased basal tension and amplitude. Ryanodine slightly increased frequency. Both iberiotoxin and NS-1619 increased amplitude. Apamin reduced frequency but increased amplitude. NS-309 inhibited both the amplitude and frequency. The basal tension and amplitude increased when H_2_O_2_ was applied. Pretreatment with NS-309 inhibited H_2_O_2_-elicited augmented amplitude and frequency, while pretreatment with Y-27632 inhibited the augmented basal tension. The combined application of NS-309 and Y27632 almost eliminated spontaneous contractions and its augmentation induced by H_2_O_2_. In conclusion, Ca^2+^ influx, Rho kinase activation, and SK channel inactivation play important roles in spontaneous contractions in intact bladder strips, whereas only latter two mechanisms may be involved in H_2_O_2_-elicited increased spontaneous contractions.

## 1. Introduction

The urinary bladder has two important functions, which are to store and expel urine. The detrusor smooth muscle of the bladder exhibits spontaneous contractile activity during the filling phase and in isolated detrusor strips [1, 2]. The spontaneous activity cannot be abolished by tetrodotoxin, atropine, phentolamine, propranolol, or hexamethonium, indicating that the activity is not dependent on innervation [1, 2]. The role of spontaneous activity may be to facilitate adjustment of muscle bundle lengths during bladder filling [3, 4]. Under certain pathological conditions, the spontaneous activity could spread and initiate synchronous contractions throughout the detrusor, generating involuntary contractions [5, 6]. Previous studies using denuded detrusor muscle strips suggested that calcium channels [7], large-conductance calcium-activated potassium [BK] channel [8], small-conductance calcium-activated potassium [SK] channel [9], Rho-associated coiled-coil kinase (ROCK) [10], and protein kinase C (PKC) [11] contributed to the regulation of smooth muscle spontaneous contractions.

Overactive bladder (OAB) is prevalent in patients or animal models with diabetes [12], bladder outlet obstruction [13], chronic ischemia [14], ischemia/reperfusion [15], or ageing [16]. These pathophysiological conditions are also characterized by excessive accumulation of reactive oxygen species (ROS). Therefore, the elevated levels of ROS have been proposed to contribute to OAB [17, 18]. More directly, we generated an inducible, smooth muscle-specific manganese superoxide dismutase (MnSOD) gene knockout mouse model recently [19]. We found the depletion of MnSOD caused oxidative stress in the bladder and the mice presented bladder overactivity [19]. Higher levels of spontaneous activity were found in detrusor muscle strips from humans and animals with OAB [5, 6]. Rabbits with chronic moderate bladder ischemia presented bladder overactivity, along with increased levels of oxidative stress markers and spontaneous bladder contractions [14]. These studies indicated the enhanced spontaneous myogenic activity might be due to higher levels of ROS.

ROS include free radicals such as superoxide anion (O_2_^•−^), hydroxyl radical (^•^OH), and nitric oxide (NO·), and nonradical molecules like peroxynitrite (ONOO−) and hydrogen peroxide (H_2_O_2_) [20, 21]. ROS have long been believed to play important roles in pathological conditions. However, recent evidence has shown that ROS may also function as a second messenger in a signaling cascade induced by changes in the ion channel activity in response to neurotransmitters and hormones [22, 23]. H_2_O_2_ is an important naturally occurring ROS and has been used to investigate the effects of ROS [24, 25]. Masuda et al. showed that intravesical instillation of H_2_O_2_ (0.003–0.3 g%) can induce bladder overactivity, including both a decrease in the intercontraction interval and an increase in the amplitude [18]. In addition, low concentrations of H_2_O_2_ have been shown to increase the contractile responses of the bladder detrusor muscle strips in a dose-dependent manner [26]. However, the effects of H_2_O_2_ on spontaneous contractions in intact bladder strips and the mechanisms involved have not been well investigated. The mucosa, including the urothelium, basement membrane, and lamina propria, can regulate detrusor contractions [27, 28]. In the present study, we investigated mechanisms of the regulation of spontaneous contractions and the effects of H_2_O_2_ in intact rat bladder strips.

## 2. Materials and Methods

### 2.1. Animals

Male Sprague-Dawley rats (10–12 weeks old, Harlan Laboratories, Indianapolis, IN) were used for this study. The animals were maintained with free access to laboratory chow and tap water in an animal facility under controlled temperature (22°C), humidity (55%  ± 10%), and lighting (12/12 h artificial light/dark cycle). The bladders were removed under 2% isoflurane anesthesia, and then animals were euthanized by a single intraperitoneal injection of pentobarbital (200 mg/kg). All protocols were approved by the Institutional Animal Care and Use Committee of Case Western Reserve University.

### 2.2. Preparation of Rat Bladder Strips and Isometric Tension Measurement

Urinary bladders were placed in cold oxygenated Krebs solution (mM: NaCl 133, KCl 4.7, NaHCO_3_ 16.3, NaH_2_PO_4_ 1.35, MgSO_4_·7H_2_O 0.6, CaCl_2_·2H_2_O 2.5, Dextrose 7.8, pH = 7.4) [29, 30]. After the surrounding adipose and connective tissues were removed, the trigone and dome area of bladders were cut and the bladder body was opened with a longitudinal incision. Full-thickness strips (8–10 mg, 2 mm in width × 10 mm in length) were prepared as we described previously [29, 30]. The strips were then mounted in a 20 ml tissue bath (Radnoti, Radnoti Glass Technology INC, Monrovia, CA) containing 20 ml Krebs solution aerated with 95% O_2_, 5% CO_2_ to obtain a pH of 7.4 at 37°C. One end of each strip was connected to a stationary glass hook, while the other end was attached to a force transducer (MLT0402, ADInstruments, Colorado Springs, CO, USA). Isometric contraction was recorded at a sampling rate of 20 Hz using a computerized data acquisition program (PowerLab 8/30 data acquisition system, ADInstruments, Colorado Springs, CO, USA) and stored on the computer for later analysis. After equilibration for 15 minutes at slack length, the strips were stretched with 1.0 gm of tension and equilibrated for another 45 minutes. During the equilibration period, the bathing solution was changed every 15 minutes and strips were slightly stretched as needed to induce the maximal spontaneous rhythmic contraction. To exclude the possible neurogenic contribution of nerves contained in the bladder strips, all experiments were performed in the presence of a neuronal inhibitory cocktail including tetrodotoxin (1 *μ*M, a neuronal Na^+^ channel blocker) and neurotransmitter receptor antagonists atropine (1 *μ*M, a muscarinic antagonist), phentolamine (1 *μ*M, an *α*-adrenergic antagonist), propranolol (1 *μ*M, a *β*-adrenergic antagonist), and suramin (10 *μ*M, a purinergic antagonist) [31, 32]. The strips, which developed less than 0.1 grams of spontaneous contractions amplitude, were excluded from further measurements.

### 2.3. Experimental Protocol and Data Analysis

After bladder strips were equilibrated, one of following test agents (specific activator or blocker) or vehicle (drug solvent) was introduced to the 20 ml bath solution and incubated for 15 minutes: nisoldipine (100 nM, an L-type voltage-gated Ca^2+^ channel blocker), SKF96365 (10 *μ*M, inhibit store-operated Ca^2+^ channel, receptor-operated Ca^2+^ channel, and voltage-operated Ca^2+^ channel), ryanodine (10 *μ*M, inhibits Ca^2+^ release from sarcoplasmic reticulum), iberiotoxin (100 nM, a BK channel blocker), NS-1619 (30 *μ*M, a BK channel activator), apamin (100 nM, a SK channel blocker), NS-309 (10 *μ*M, a SK channel activator), bisindolylmaleimide 1 (BIS1, 2 *μ*M, a protein kinase C inhibitor), or Y27632 (10 *μ*M, a ROCK inhibitor). The concentrations of the specific blockers or activators were chosen based on the previous studies [31–33]. Then, 20 *μ*l of 3 g% (grams/100 ml) H_2_O_2_ was added to the bath solution (the final concentration of H_2_O_2_ was 0.003 g%) and incubated for 15 min. The concentration of H_2_O_2_ was chosen based on the previous in vivo [18] and in vitro [25, 26, 34] studies. In addition, our own pilot experiment showed 0.003 g% H_2_O_2_ can induce obvious augmentation of spontaneous contractions. Data were collected using a PowerLab 8/30 data acquisition system and LabChart 6 Pro (Version 6.1.1) software (ADInstruments, Colorado Springs, CO, USA).

**Figure 1:**
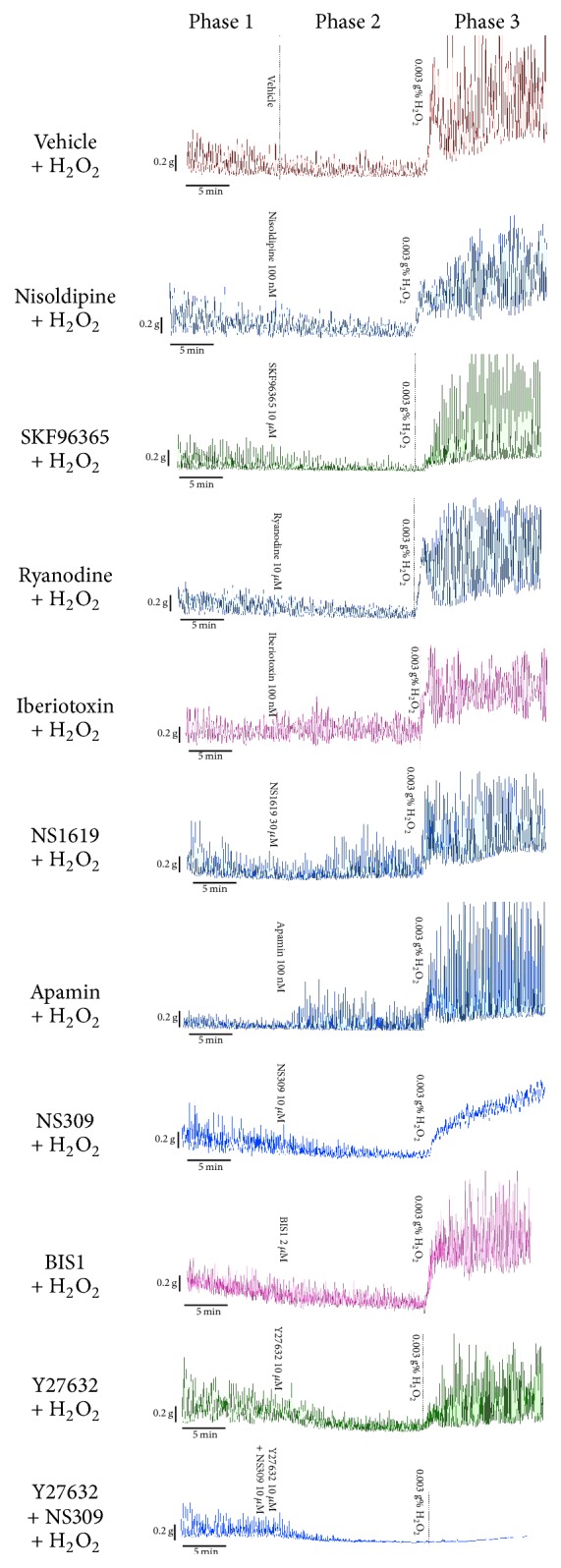
Representative tracings of spontaneous contractions of intact rat bladder strips before (Phase 1) and during (Phase 2) treatment with vehicle (0.1% DMSO) or test agent [nisoldipine (100 nM), SKF96365 (10 *μ*M), ryanodine (10 *μ*M), iberiotoxin (100 nM), NS1619 (30 *μ*M), apamin (100 nM), NS309 (10 *μ*M), Y27632 (10 *μ*M), BIS-1 (2 *μ*M), and Y27632 + NS309 (each 10 *μ*M)] and then incubated with 0.003 g% H_2_O_2_ (Phase 3).

**Figure 2:**
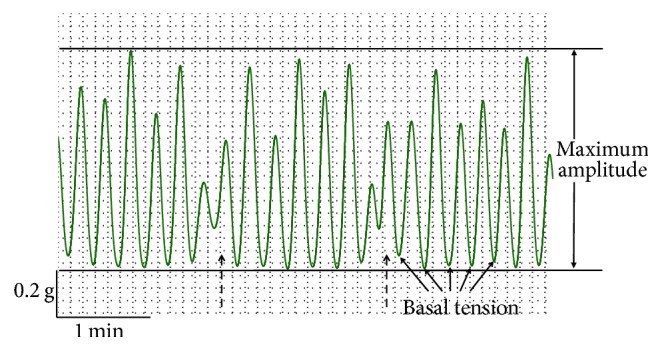
A zoomed image of spontaneous contraction tracings illustrating the quantified parameters. The spontaneous contractile events, the amplitude of which exceed 30% of the maximum response for the final 5 min in each 15 min incubation period, were used for analysis. For quantification of frequency, the contraction with multiple peaks due to partial relaxation (shown as a dashed arrow) was counted as one contraction event.

The timeline of the experimental protocol can be seen in Figure 1, which shows representative tracings of spontaneous contractions in bladder strips. For analysis, the tracings were divided into three sections according to the time of application of the different agents: Phase 1 is the period after application of the neuronal inhibitory cocktail only, Phase 2 is the period of incubation of the bladder strip with a test agent or vehicle, and Phase 3 is the period after addition of H_2_O_2_ to the bath containing inhibitory cocktail and test agent or vehicle. The responses during the final 5 min of each 15 min incubation period in different phases were used for analysis. Three parameters of spontaneous contractions, basal tension (the average of the lowest tension of phasic spontaneous contractions), amplitude (the average amplitude of spontaneous contractions), and frequency (number of contractions per minute), were measured using LabChart 6 Pro software.  Figure 2 illustrated how the parameters were measured. The spontaneous contractile events, the amplitude of which exceed 30% of the maximum response for the final 5 min in each 15 min incubation period, were used for analysis as previously described [32]. In addition, the contraction with multiple peaks due to partially relaxation was counted as one contraction event.

The effects of a test agent on spontaneous contractions were determined by calculating the percentages of Phase 2 values to Phase 1 values when the test agent was applied in Phase 2 and comparing those percentages to the corresponding percentages when Phase 2 incubation was with vehicle alone. Similarly, the effects of a test agent on H_2_O_2_-induced altered spontaneous contractions were determined by comparing the percentages of Phase 3 values to Phase 1 values when the test agent was present in Phase 2 to the corresponding percentages in the absence of the test agent.

### 2.4. Drugs and Solutions

Tetrodotoxin, phentolamine, iberiotoxin, NS-1619, Y-27632, apamin, NS-309, and ryanodine were purchased from Abcam (Cambridge, MA, USA). Atropine, propranolol, suramin, nisoldipine, and SKF-96365 were purchased from Sigma-Aldrich (St. Louis, MO USA). Bisindolylmaleimide 1 and H_2_O_2_ were obtained from Cayman Chemical (Ann Arbor, MI, USA) and Fisher Scientific (Pittsburg, PA, USA), respectively.

NS-1619, nisoldipine, NS 309, and SKF-96365 were dissolved in dimethylsulphoxide (DMSO). Ryanodine was dissolved in 100% ethanol. All other drugs were prepared with water. Final ethanol and DMSO concentrations in the bath solution did not exceed 0.1% and were shown not to affect spontaneous contractions or bladder function in previous reports [35–37].

### 2.5. Statistical Analysis

Statistical analysis was performed using GraphPad Prism 6 (GraphPad Software, La Jolla, CA). The data are presented as mean ± SEM. For comparison between vehicle and drug application group, unpaired nonparametric Mann–Whitney test was used. A *p* value less than 0.05 was considered statistically significant.

## 3. Results

### 3.1. Rhythmic Spontaneous Contractions in Intact Bladder Strips Were Observed

As shown in Figure 1, rhythmic spontaneous contractions occurred in intact bladder strips in the presence of the neuronal inhibitory cocktail, suggesting a myogenic basis of the contraction. However, the basal tension and the contraction amplitude decreased within the investigated period. In the control (vehicle-treated) group (the data from water, ethanol, and DMSO-treated strips were pooled since less than 0.1% ethanol or DMSO did not affect spontaneous contractions in previous reports [35–37] and in our pilot study), the basal tension and amplitude decreased 13.6% and 32.5%, respectively, from Phase 1 to Phase 2, while the frequency did not change significantly (Figure 1, Table 1).

### 3.2. Roles of Calcium Channels on Spontaneous Contractions in Intact Bladder Strips

Nisoldipine had no significant effect on spontaneous contractions of intact bladder strips (Figure 1, Table 1), whereas SKF96365 decreased the amplitude dramatically and basal tension slightly (Figure 1, Table 1). Application of ryanodine resulted in an increase in the frequency, but no significant change in the basal tension and amplitude (Figure 1, Table 1).

### 3.3. Roles of Potassium Channels on Spontaneous Contractions in Intact Bladder Strips.

Iberiotoxin increased the basal tension and the amplitude of spontaneous contractions slightly, but did not affect frequency (Figure 1, Table 1). Interestingly, NS-1619 increased contraction amplitude, as well as basal tension significantly (Figure 1, Table 1). Apamin reduced frequency and increased amplitude dramatically but did not affect basal tension (Figure 1, Table 1). NS-309 markedly inhibited both the amplitude and frequency of spontaneous contractions but caused very little change in basal tension (Figure 1, Table 1).

### 3.4. Roles of Ca^2+^ Sensitivity-Related Protein Kinases on Spontaneous Contractions in Intact Bladder Strips

To study the roles of Ca^2+^ sensitivity-related protein kinases ROCK, and PKC in spontaneous contractions in bladder strips, PKC inhibitor bisindolylmaleimide I (BIS1) and the ROCK inhibitor Y27632 were used. As shown in Figure 1 and Table 1, BIS1 (2 *μ*M) did not affect the basal tension, amplitude, or frequency of spontaneous contractions in intact bladder strips. Y27632 (10 *μ*M) significantly inhibited the basal tension and amplitude of the contractions, whereas the frequency was increased.

### 3.5. Effects of Combined Application of ROCK Inhibitor and SK Channels Activator on Spontaneous Contractions in Intact Bladder Strips

Based on our observation that NS-309 can inhibit the amplitude and frequency of spontaneous contractions and Y27632 can inhibit the basal tension and amplitude of the contractions, we expected that the combined application of NS-309 and Y27632 would eliminate the spontaneous contractions. Indeed, the combined application of NS-309 and Y27632 eliminated spontaneous contractions almost completely (Figure 1, Table 1).

### 3.6. H_2_O_2_ Induced Augmentation of Spontaneous Contractions in Intact Bladder Strips

The basal tension and amplitude of spontaneous contractions increased significantly in the vehicle-pretreated group when 0.003% H_2_O_2_ was applied. Basal tension increased to 204.6% and amplitude increased to 216.4% of Phase 1, whereas frequency was not affected (Figure 1 and Table 2).

### 3.7. Roles of Calcium Channels in the H_2_O_2_-Induced Augmentation of Spontaneous Contractions in Intact Bladder Strips

As shown in Figure 1 and Table 2, pretreatment with nisoldipine (100 nM), SKF-96365 (10 *μ*M), or ryanodine (10 *μ*M) did not prevent the increases in basal tension and amplitude of spontaneous contractions induced by 0.003 g% H_2_O_2_ and did not affect the frequency significantly compared to the vehicle-treated group.

### 3.8. Roles of Potassium Channels in the H_2_O_2_-Induced Augmentation of Spontaneous Contractions in Intact Bladder Strips

Neither the BK channel blocker iberiotoxin (100 nM) nor the BK channel activator NS-1619 (30 *μ*M) affected the H_2_O_2_-induced augmentation of spontaneous contractions (Figure 1, Table 2). Pretreatment with the SK channel blocker apamin (100 nM) did not affect the H_2_O_2_-induced increase of the basal tension; however, it dramatically enhanced the effect of H_2_O_2_ on the amplitude (Figure 1, Table 2). The SK channel activator NS-309 (10 *μ*M) significantly prevented the increase in the amplitude of spontaneous contractions induced by H_2_O_2_ and caused a dramatic reduction in frequency. However NS-309 did not affect the H_2_O_2_-induced increase of the basal tension.

### 3.9. Roles of Ca^2+^ Sensitivity-Related Protein Kinases on the H_2_O_2_-Induced Augmentation of Spontaneous Contractions in Intact Bladder Strips

As illustrated in Figure 1, the PKC inhibitor BIS1 (2 *μ*M) had no effect on the H_2_O_2_-induced augmentation of spontaneous contractions. The ROCK inhibitor Y-27632 (10 *μ*M) markedly prevented the H_2_O_2_-elicited augmentation of basal tension and increased frequency slightly. However it did not attenuate the augmented amplitude induced by H_2_O_2_ (Figure 1, Table 2).

### 3.10. Effects of Combined Application of ROCK Inhibitor and SK Channels Activator on the H_2_O_2_-Induced Augmentation of Spontaneous Contractions in Intact Bladder Strips

Upon observing that NS-309 can decrease the amplitude and frequency of the H_2_O_2_-induced augmentation of spontaneous contractions and Y27632 can inhibit the basal tension of the augmented contractions, we postulated that the combined application of NS-309 and Y27632 would affect the H_2_O_2_-induced augmented spontaneous contractions. Indeed, the combined application of NS-309 and Y27632 significantly prevented the H_2_O_2_-induced augmentation of spontaneous contractions, particularly the amplitude (Figure 1, Table 2).

## 4. Discussion

In order to closely reflect the in vivo situation, we chose to use intact full-thickness bladder strips in this study instead of detrusor muscle layer only, which were used in most previous studies [32, 38]. The spontaneous contractions may increase [39] or decrease [27] in intact bladder strips compared to the bladder smooth muscle only strips. Current studies demonstrated that rhythmic spontaneous contractions occurred in intact bladder strips and persisted in the presence of a neuronal inhibitory cocktail. The frequency of spontaneous contractions did not change. The basal tension and the amplitude decreased slightly over the investigated period, which is probably due to stress relaxation.

It is well established that contraction of smooth muscle such as detrusor is modulated primarily by the intracellular Ca^2+^ concentration [40]. Calcium influx and calcium release from sarcoplasmic reticulum have been indicated to play important roles in regulating smooth muscle contraction [40]. In the present study, SKF96365, which inhibits the calcium entry through store-operated Ca^2+^ channel, receptor-operated Ca^2+^ channel, and voltage-operated Ca^2+^ channel [41], can significantly inhibit spontaneous activity in intact bladder strips, indicating Ca^2+^ influx is involved. However, L-type voltage-gated Ca^2+^ channel blocker nisoldipine, which has been shown to inhibit spontaneous contractions in bladder detrusor muscle [27, 32], did not affect spontaneous activity in intact bladder strips. In addition, the ryanodine (10 *μ*M), which was shown inhibiting ryanodine receptor-mediated Ca^2+^ release and suppressing spontaneous activity of the bladder detrusor muscle [27, 33], slightly increases the frequency of intact bladder strips. Therefore, current results in intact bladder strips are not fully consistent with those previously reported in detrusor muscle only [27]. It looks that mucosa, which consists of the urothelium, basement membrane, and lamina propria [28], may play an role in regulation of the spontaneous contractions. Mucosa may weaken or offset the effects of nisoldipine or ryanodine on spontaneous contractions and therefore maintain the amplitude in intact bladder strips. Urothelial cells express a variety of receptors and ion channels and can release diffusible agents, for example, prostaglandins, which could enhance spontaneous activity in the intact bladder [28, 42, 43]. The different results also could be due to the different species used. Guinea-pig and pig detrusor strips were used in the above-mentioned studies [27, 32, 33]. Another possible reason is that we only examined the effects of a single dose of nisoldipine (100 nM) [27] and ryanodine (10 *μ*M) on spontaneous contractions. The effects of different doses need to be examined in the future. Different doses of activators/blockers may have different effects on Ca^2+^ influx and release. Ryanodine has been reported to promote Ca^2+^ release at low concentrations, while locking up the ryanodine sensitive channels and reducing or preventing Ca^2+^ release at high concentrations [44].

Although all three subtypes of calcium-activated potassium channels (K_(Ca)_) including large, intermediate, and small-conductance K_(Ca)_ (BK, IK, and SK) are present in the bladder, previous study suggested that IK is not important in regulation of bladder contraction [9], whereas the opening of BK and SK channels has been shown to cause detrusor smooth muscle relaxation in human [45] and rat [46] through the leak of potassium ions along their concentration gradient into the extracellular space. The current study showed that blocking BK channel with iberiotoxin slightly increased contraction amplitude of the intact bladder strip, which is the same as the results from the detrusor smooth muscle only strip. Surprisingly, BK channel activator NS1619 (30 *μ*M) also increases the basal tension and amplitude in intact bladder strips. This is contrary to the previous studies performed in bladder detrusor muscle strips [47, 48]. The phenomenon may be due to the presence of mucosa. Although BK channels are mainly located in detrusor smooth muscle cells, several studies showed that mucosa also express BK channels [49, 50]. The function of BK channels in urothelium is not clear. Blocking SK channels with apamin reduced contraction frequency and increased the contraction amplitude, while SK channel opener NS309 decreased the spontaneous contractions amplitude and frequency significantly in intact bladder strips. These results are consistent with the studies performed in detrusor muscle strips [31, 38].

In addition to intracellular Ca^2+^ concentration, Ca^2+^ sensitization also plays an important role in smooth muscle contraction [40]. ROCK and PKC are two major molecules that regulate Ca^2+^ sensitization [51]. ROCK inhibitor Y27632 decreased both the amplitude and basal tension but increased the frequency of spontaneous contractions in intact bladder strips. This result indicated that ROCK activation plays an important role in spontaneous contractions, whereas PKC inhibitor BIS1 did not suppress the spontaneous contractile amplitude, frequency, and basal tension, suggesting that PKC pathway may not play an important role in mediating spontaneous contractions in intact bladder strips. The data support a previous study [10] which showed that the ROCK inhibitors (1 *μ*M and 3 *μ*M Y-27632 and 10 *μ*M HA-1077) reduced both basal myosin light chain phosphorylation and tone in rabbit detrusor at the optimum length for muscle contraction, whereas the PKC inhibitor (1 *μ*M GF-109203X) did not produce a significant reduction in myosin light chain phosphorylation and tone. However, the results are not consistent with another study which showed PKC inhibitor helped to maintain the amplitude of spontaneous contractions of rabbit detrusor smooth muscle strips [11]. In addition to inhibiting the amplitude of spontaneous contractions, NS309 inhibits the frequency, whereas Y27632 suppresses the base tension. Therefore, we expected that when NS309 and Y27632 were used together, the overall inhibitory response would be enhanced. We found indeed that the application of NS309 and Y27632 together almost eliminated the spontaneous contractions in intact bladder strips.

A previous study showed that H_2_O_2_ can induce contraction in rat detrusor smooth muscle strips [26]. Our interest was to determine the effects of H_2_O_2_ on the spontaneous contractions and the underlying mechanisms in intact bladder strips. We found H_2_O_2_ can increase the basal tension and amplitude, but not the frequency of spontaneous contractile activity in intact bladder strips. The increased amplitude may be due to direct effects or indirect effects through increasing basal detrusor smooth muscle tone. Pretreatment with nisoldipine, SKF96365, and ryanodine did not prevent H_2_O_2_-induced augmentation of the basal tension and amplitude of spontaneous contractions, which suggests Ca^2+^ influx and ryanodine receptor-related Ca^2+^ release from sarcoplasmic reticulum are not essential to the H_2_O_2_-induced increase of spontaneous contractions in intact bladder strips. In addition, pretreatment with BK channel activator NS-1619 or blocker iberiotoxin and PKC inhibitor Bis1 did not affect H_2_O_2_-elicited increases of the basal tension and amplitude. However, SK channel activator NS-309 pretreatment significantly inhibited the H_2_O_2_-elicited increase of amplitude, and SK channel blocker apamin pretreatment enhanced the H_2_O_2_-elicited increase of the amplitude. These results indicated that H_2_O_2_ increased the amplitude of spontaneous contractions at least partially through the deactivation of SK channel. H_2_O_2_ regulates the activity of the proteins either through direct oxidative modification or indirectly through modification of associated signaling molecules [52]. Wei et al. used the patch-clamp technique to study the effect of H_2_O_2_ on SK channel in the cortical collecting duct. They found the addition of H_2_O_2_ decreased the activity of the SK channel by stimulating protein tyrosine kinase, P38, and ERK in the cortical collecting duct and then enhancing the internalization of the SK channel [53]. In addition, ROCK inhibitor Y-27632 significantly inhibited the H_2_O_2_-elicited increase of basal tension, suggesting that H_2_O_2_ may increase Ca^2+^ sensitivity by regulation of the ROCK pathway. Multiple studies have demonstrated that H_2_O_2_ can enhance the activation of ROCK and mediate vascular contraction [54, 55]. In spontaneously hypertensive rats, increased ROCK activation was involved in the H_2_O_2_-induced contraction of mesenteric resistance arteries [55]. H_2_O_2_ activates ROCK possibly through the activation of the upstream molecule, RhoA, but not direct activation of ROCK, since ROCK is not directly redox sensitive [56]. Considering the different effects of NS309 and Y27632 on the H_2_O_2_-induced augmentation of spontaneous contractions, we postulated that they might have synergistic effects when they were applied together. The results actually showed that combined application of Y-27632 and NS-309 significantly prevented the H_2_O_2_-induced increases of basal tension, amplitude, and frequency of the spontaneous contractions.

## 5. Conclusions

Our results suggest that Ca^2+^ influx, Rho kinase activation, and SK channel deactivation may play important roles in the spontaneous contractions in intact bladder strips, whereas only latter two mechanisms may be involved in H_2_O_2_-induced augmentation of spontaneous contractions. Previous studies indicated that excessive accumulation of ROS [17–19, 57] and enhanced spontaneous contractions [5, 6, 14] are related to OAB, indicating high levels of ROS may contribute to the enhanced spontaneous contractions. The current study explored the potential mechanisms of the H_2_O_2_-induced augmentation of spontaneous contractions through pharmacological approach. One limitation is that we used just a single dose of activator/blocker based on the previous reports. Dose-response effects of the specific activators/blockers on H_2_O_2_ induced augmentation of spontaneous contractions need to be investigated. In addition, future experiments are warranted to (1) confirm the current results using electrophysiological techniques; (2) determine the effects of combined application of SK channel activator and ROCK inhibitor on ROS-related OAB in animal models.

## Figures and Tables

**Table 1:** Effects of specific activators and blockers on spontaneous contractions in intact bladder strips (Phase 2 values as percentages of Phase 1 values).

Drugs (concentration)	*n*	Basal tension (%)	Amplitude (%)	Frequency (%)
Control (vehicle + H_2_O_2_)	13	86.4 ± 2.36	67.5 ± 5.09	103.4 ± 3.29
Nisoldipine (100 nM)	6	79.5 ± 4.86	67.8 ± 7.73	103.3 ± 3.41
SKF96365 (10 *µ*M)	6	75.4 ± 3.93^*∗*^	30.7 ± 4.48^*∗*^	100.7 ± 7.31
Ryanodine (10 *µ*M)	5	89.8 ± 8.20	82.4 ± 8.89	124.8 ± 8.57^*∗*^
Iberiotoxin (100 nM)	8	96.8 ± 3.99^*∗*^	92.4 ± 4.31^*∗*^	106.8 ± 4.43
NS1619 (30 *µ*M)	5	101.8 ± 6.07^*∗*^	152.4 ± 14.19^*∗*^	109.2 ± 5.97
Apamin (100 nM)	9	84.7 ± 5.64	325.3 ± 73.87^*∗*^	82.2 ± 5.32^*∗*^
NS309 (10 *µ*M)	6	80.1 ± 2.20	41.4 ± 6.44^*∗*^	61.1 ± 6.36^*∗*^
BIS1 (2 *µ*M)	7	81.0 ± 2.45	68.78 ± 4.97	118.4 ± 16.58
Y27632 (10 *µ*M)	9	59.9 ± 4.71^*∗*^	45.7 ± 2.02^*∗*^	131.6 ± 7.53^*∗*^
Y27632 (10 *µ*M) + NS309 (10 *µ*M)	5	50.9 ± 1.19^*∗*^	3.3 ± 3.34^*∗*^	14.7 ± 14.71^*∗*^

Values are expressed as mean plus or minus SEM of 5 to 13 individual strips; ^*∗*^significantly different from corresponding value in vehicle treated group (*p* < 0.05).

**Table 2:** Effects of specific activators and blockers on H_2_O_2_-induced augmentation of spontaneous contractions in intact bladder strips (Phase 3 values as percentages of Phase 1 values).

Drugs	*n*	Basal tension (%)	Amplitude (%)	Frequency (%)
Control (vehicle + H_2_O_2_)	13	204.6 ± 10.79	216.4 ± 22.75	100.7 ± 3.00
Nisoldipine + H_2_O_2_	6	195.0 ± 14.81	236.9 ± 47.62	105.8 ± 5.25
SKF96365 + H_2_O_2_	6	174.2 ± 8.85	246.4 ± 56.27	101.5 ± 4.95
Ryanodine + H_2_O_2_	5	209.0 ± 20.37	253.8 ± 52.82	130.4 ± 13.43
Iberiotoxin + H_2_O_2_	8	238.8 ± 13.63	339.3 ± 57.82	96.6 ± 8.39
NS1619 + H_2_O_2_	5	248.3 ± 16.42	318.1 ± 55.97	98.8 ± 7.57
Apamin + H_2_O_2_	9	222.5 ± 13.48	1025.7 ± 217.71^*∗*^	84.2 ± 7.39
NS309 + H_2_O_2_	6	208.5 ± 25.43	84.49 ± 13.55^*∗*^	41.2 ± 5.46^*∗*^
BIS1 + H_2_O_2_	7	211.0 ± 22.01	230.0 ± 31.13	104.9 ± 9.13
Y27632 + H_2_O_2_	9	128.7 ± 17.98^*∗*^	195.4 ± 35.64	125.3 ± 8.51^*∗*^
Y27632 + NS309 + H_2_O_2_	5	97.4 ± 4.72^*∗*^	9.4 ± 3.97^*∗*^	31.6 ± 19.23^*∗*^

Values are expressed as mean plus or minus SEM of 5 to 13 individual strips; ^*∗*^significantly different from corresponding value in vehicle treated group (*p* < 0.05).
